# Site-specific detection of protein secondary structure using 2D IR dihedral indexing: a proposed assembly mechanism of oligomeric hIAPP†
†Electronic supplementary information (ESI) available: Expanded view of 2D IR spectra, ThT fluorescence measurements, TEM images. Subtraction of the TRIS buffer. Diagonal slices of all the studied labels in TFE/TRIS 50%/50% (v/v) mixture. Diagonal cuts through the fundamental band of all the labels at different aggregation times. NNMF analysis of only the isotope region of L12A13 showing three-state kinetics. See DOI: 10.1039/c7sc03789a


**DOI:** 10.1039/c7sc03789a

**Published:** 2017-11-03

**Authors:** Michał Maj, Justin P. Lomont, Kacie L. Rich, Ariel M. Alperstein, Martin T. Zanni

**Affiliations:** a Department of Chemistry , University of Wisconsin-Madison , Madison , Wisconsin 53706-1396 , USA . Email: zanni@chem.wisc.edu

## Abstract

Human islet amyloid polypeptide (hIAPP) aggregates into fibrils through oligomers that have been postulated to contain α-helices as well as β-sheets. We employ a site-specific isotope labeling strategy that is capable of detecting changes in dihedral angles when used in conjunction with 2D IR spectroscopy. The method is analogous to the chemical shift index used in NMR spectroscopy for assigning protein secondary structure. We introduce isotope labels at two neighbouring residues, which results in an increased intensity and positive frequency shift if those residues are α-helical *versus* a negative frequency shift in β-sheets and turns. The 2D IR dihedral index approach is demonstrated for hIAPP in micelles for which the polypeptide structure is known, using pairs of ^13^C^18^O isotope labels L12A13 and L16V17, along with single labeled control experiments. Applying the approach to aggregation experiments performed in buffer, we show that about 27–38% of hIAPP peptides adopt an α-helix secondary structure in the monomeric state at L12A13, prior to aggregation, but not at L16V17 residues. At L16V17, the kinetics are described solely by the monomer and fiber conformations, but at L12A13 the kinetics exhibit a third state that is created by an oligomeric intermediate. Control experiments performed with a single isotope label at A13 exhibit two-state kinetics, indicating that a previously unknown change in dihedral angle occurs at L12A13 as hIAPP transitions from the intermediate to fiber structures. We propose a mechanism for aggregation, in which helices seed oligomer formation *via* structures analogous to leucine rich repeat proteins.

## Abstract 2

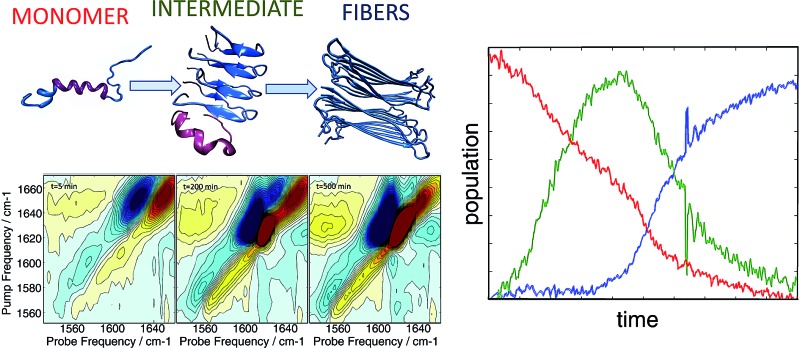
Human islet amyloid polypeptide (hIAPP) aggregates into fibrils through oligomers that have been postulated to contain α-helices as well as β-sheets.

## A Introduction

The abnormal aggregation of peptides and proteins into amyloid fibrils is associated with more than 20 human diseases, which include metabolic disorders like type 2 diabetes^1^ as well as various neurodegenerative disorders such as Alzheimer's and Parkinson's diseases.^2^ It has been found that each amyloid disease is associated with a different protein. We focus our present study on the human islet amyloid polypeptide (hIAPP or amylin) whose aggregation is thought to cause the loss of the insulin-producing β-cells in type 2 diabetes mellitus.^3^ The number of adults with type 2 diabetes was estimated to be 347 million in 2011 and has been dramatically rising since then.^4^

Most amyloid fibrils adopt the so-called cross-β architecture in which the extended, intermolecular β-sheets stack together with their β-strands aligned perpendicularly to the fibril axis.^5^ For type 2 diabetes it is probably not the fibrils, but the transient intermediates, that are responsible for the cytotoxicity of hIAPP to pancreatic β-cells.^6^–^9^ Presumably, the oligomers adopt a structure that is toxic when interacting with membranes or specific proteins. Therefore, it is important to provide a molecular description of the changes occurring at each stage of the aggregation process on the way to the formation of amyloid fibrils.

hIAPP aggregation most likely involves α-helical structures.^10^–^12^ hIAPP forms α-helices when it interacts with negatively-charged membranes.^11^,^13^–^15^ CD spectra of the membrane-bound hIAPP show that 69% of the polypeptides are bound to the membrane and that each bound peptide contains 50–55% of residues in a helical conformation. Electron paramagnetic resonance (EPR) have also detected α-helical conformations of the hIAPP immersed in lipid bilayers.^16^,^17^ Negatively charged membranes accelerate aggregation, which has led to the hypothesis that the observed α-helical fragments nucleate aggregation into larger α-helical assemblies which, subsequently, convert cooperatively to amyloid fibers.^10^,^13^,^14^ The role of α-helices for IAPP aggregation in solution, on the other hand, is less clear. According to molecular dynamics simulations and solution NMR measurements, the monomer contains α-helices in the N-terminus, which is stabilized by the disulfide bridge between Cys2 and Cys7.^18^ De Carufel *et al.* have suggested, based on CD results on a helix-disrupting D-hIAPP analog, that α-helices are off-pathway steps to fiber formation in the aggregation mechanism and that helices may actually reduce IAPP-induced cytotoxicity.^19^

Because the α-helices and intermediates are transient, many studies purposefully slow aggregation using low pH buffers, low temperature, or chemical modifications.^11^,^14^,^16^–^18^,^21^ While slow aggregation makes aggregation easier to study, these conditions may alter the native structure of hIAPP. Most noteworthy, at low pH the His-18 residue becomes protonated which, consequently, creates strong repulsive forces between charged histidines, affecting the interaction between the histidine and the C-terminus tyrosine, and resulting in amyloid fibers of significantly different morphology.^22^–^24^ Moreover, CD spectroscopy has shown that pH and temperature can generally affect the population of α-helical structures in a variety of different peptides.^25^,^26^ Therefore, the ability to obtain detailed information on the molecular structure of dynamically evolving species, such as intermediates of hIAPP, without perturbative conditions or non-natural mutations is highly desirable.

**Fig. 1:**
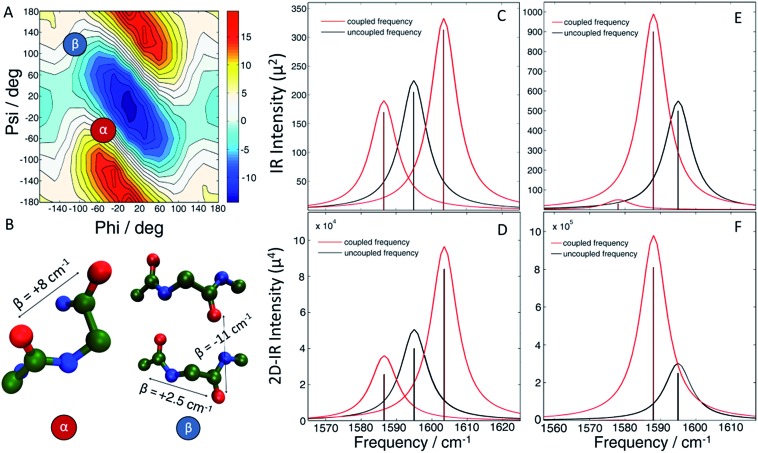
(A) Amide I frequency map^20^ plotted as a function of Ramachandran angles. (B) Model structure of an α-helix (left) showing positive coupling between adjacent amino acids and a β-sheet (right) which exhibits positive couplings between nearest-neighbours as well as a strong inter-strand coupling, both contributing to a negative frequency shift. (C and D) The resulting (C) linear and (D) diagonal peaks of a 2D IR spectrum of the α-helix model. (E and F) The resulting (E) linear and (F) diagonal peaks of a 2D IR spectrum of the β-sheet model.

In the present work, we apply two-dimensional IR (2D IR) spectroscopy^27^–^29^ to measure aggregation kinetics. The spectrometer uses a pulse shaper that generates a new pulse train shot-to-shot, enabling rapid “on the fly” spectral acquisition. 2D IR spectroscopy has been previously used for studying aggregation kinetics of amyloid polypeptides.^30^–^32^ Indeed, we have previously observed 2D IR features consistent with α-helices in experiments on hIAPP associated with membrane bilayers.^15^ However, those experiments did not determine the location of the helices in the polypeptide. To obtain residue-specific structural and kinetic information, isotope labelling is needed, such as ^13^C^18^O labelling an individual amino acid. When an isotope label becomes incorporated into a parallel β-sheet, like many residues do when fibers form, coupling between strands produce a negative shift in the labelling frequency.^32^ Thus, single labels are very sensitive to amyloid β-sheet formation. In contrast, a single isotope label is largely insensitive to the formation of α-helices, because there are no couplings to other labels that would cause a frequency shift. The strategy presented here, which we call 2D IR dihedral indexing, is based on labeling two neighbouring residues to take advantage of vibrational coupling constants that depend on dihedral angles. Dihedral angles are monitored by frequency shifts and intensity changes. Shown in Fig. 1A is a plot of the coupling between adjacent residues as a function of the dihedral angles *ψ* and *φ*. Coupling causes the vibrational modes of the isotope labels to coherently oscillate, producing splitting and frequency shifts, such as illustrated in Fig. 1 for simulated FTIR spectra and the diagonal peaks of 2D IR spectra. The coupling constant between residues adopting the dihedral angles of an α-helix is +8.3 cm^–1^ (Fig. 1A red dot, Fig. 1B shows the structure). Because the carbonyl groups between two adjacent amino acids are nearly parallel, the positive coupling constants of an α-helix will produce two peaks with the more intense peak at a higher frequency (Fig. 1C). In a β-sheet conformation, the coupling between nearest-neighbour residues is +2.5 cm^–1^ (Fig. 1A blue dot, Fig. 1B has structure), but because the carbonyl groups on two adjacent residues are anti-parallel, the more intense peak is at a lower frequency (Fig. 1E). In addition to the intra-strand coupling, there is also the inter-strand coupling between residues on different strands in a β-sheet. As stated above, the inter-strand coupling for in-register residues of a parallel β-sheet, such as forms in hIAPP fibers, is negative (Fig. 1B). Since the in-register carbonyls are parallel, the negative coupling also causes a negative frequency shift.^33^ Thus, both intra- and inter-strand couplings for β-sheets contribute to a negative frequency shift whereas an α-helix has a positive frequency shift. Intensity changes caused by coupling are even more apparent in 2D IR than FTIR spectra. Coupling redistributes the oscillator strengths, which are quantified as the transition dipole strengths, *μ*. 2D IR signals scale as |*μ*|^4^ whereas FTIR signals scale as |*μ*|^2^. Thus, 2D IR is much more sensitive than FTIR to structural changes. Spectra of singly isotope labelled peptides are used for control experiments to separate vibrational shifts due to a change in electrostatic environment from shifts caused by vibrational coupling.^32^–^35^ Thus, double labels provide a qualitative and quantitative measure of the secondary structure, with a positive frequency shift indicating an α-helix and a negative shift a β-sheet, and an intensity enhancement over random coils. Helices might also be detected by couplings between hydrogen bonded isotope labels at *n* and *n* + 3 residues, but the frequency shift would be in the same direction as β-sheets.^36^,^37^ Nearest neighbour *n* and *n* + 1 labels give opposite shifts. FTIR spectroscopy might be used instead of 2D IR,^38^,^39^ but FTIR lacks the intensity enhancement and accompanying background suppression of 2D IR spectroscopy. Because the frequency shifts are created by dihedral angles, the strategy outlined here can be applied to any protein system. It is analogous to NMR chemical shift index (CMI) measurements for assigning secondary structure, hence our choice of name, 2D IR dihedral indexing.^40^ For an amyloid system, inter-strand coupling magnifies the negative frequency shift. In this paper, we establish the coupling scheme for dihedral indexing using hIAPP in micelles and then apply it to study the conformation kinetics of hIAPP during aggregation into amyloid fibers at the L12A13 and L16V17 sites. Three-state aggregation kinetics are observed at L12A13, corresponding to a structural transition from the monomer to an oligomeric intermediate, and then to the β-sheets of the amyloid fiber, thereby resolving a previously unknown structural transition in the N-terminus. Thus, the oligomeric intermediate contains at least two regions of hierarchical structure, at the L12A13 region and in the previously reported FGAIL region. We propose a structure, based on a known protein fold, for the oligomer that appears consistent with many experimental observations.

## B Materials and methods

### Peptide synthesis and purification

Isotope-labeled hIAPP was synthesized by Fmoc solid-phase peptide synthesis on PAL–PEG–PS resin according to published protocols.^41^,^42^ Synthesized peptides were cleaved off resin with a microwave-assisted cleavage system (Accent, CEM) using TFA : H_2_O : TIS (18 : 1 : 1, v/v/v) mixture. Crude peptide was dissolved in dimethyl sulfoxide (DMSO) for 24 hours to form a disulfide bond between Cys2 and Cys7. The oxidized peptide was then purified with reversed-phase HPLC using C18 preparative column (Waters XSelect). Buffer A consisted of 0.045% HCl in H_2_O and buffer B of 80% acetonitrile, 20% water and 0.045% HCl (v/v). Purification was run with a gradient of 1% buffer B per minute. All measurements were carried out at 1 mM peptide concentration.

### 2D IR spectroscopy

The 2D IR experimental setup consists of a one-box ultrafast amplifier (Solstice, Spectra-Physics) and a commercial BBO-based optical parametric amplifier (TOPAS, Light Conversion Ltd.). The signal and idler beams generated from the OPA are focused onto a difference-frequency generation crystal (AgGaS_2_) to yield 6 μm mid-IR pulse, subsequently split into pump and probe pulses. The pump beam is guided through a germanium acousto-optic modulator (AOM)-based pulse shaper, enabling shot-to-shot delay scanning and phase cycling.^43^ The beams are then focused onto the sample and the self-heterodyned signal is measured with a nitrogen-cooled MCT array detector. At the 1 kHz repetition rate of this laser system, it takes 424 ms to collect a 2D IR spectrum. For kinetics measurements, 2D IR spectra are collected continuously, creating data sets of thousands of 2D IR spectra to which a running average is applied to achieve the desired signal-to-noise. A more detailed description of pulse-shaping assisted 2D IR spectroscopy has been given in detail elsewhere.^44^–^47^

### IR spectra simulations and data analysis

Linear and nonlinear IR spectra of a coupled oscillator system were calculated using the exciton method^48^ in which the diagonal elements are the local frequencies in the absence of vibrational coupling and off-diagonal elements are coupling constants between the singly excited states. To simulate 2D IR spectra one needs to consider a three-level system with two-exciton diagonal Hamiltonian elements shifted by anharmonicity. An anharmonicity of 16 cm^–1^ is typically chosen for amide I transitions. The coupling constants were taken from the Jansen's frequency map^20^ for neighbouring residues and estimated from transition dipole coupling (TDC) for other coupling elements. Dipole moments in TDC calculations have a magnitude of 3.7 D Å^–1^ amu^1/2^ and are placed on each carbonyl group at an angle of 20° from its axis. Diagonalized Hamiltonian matrices yield eigenvalues and eigenvectors that determine the frequency and intensity of vibrationally coupled states.

Time-resolved 2D IR spectra presented in this work were analyzed with a non-negative matrix factorization algorithm.^49^ The algorithm uses an input matrix ***I*** consisting of time-resolved diagonal traces extracted from the 2D spectra. The traces are fit to obtain a set of ***W*** matrices, which contain vibrational eigenspectra, and ***H*** matrices, which are the time-dependent intensity changes. Matrix multiplication of the ***W*** and ***H*** matrices is equal to the input matrix minus the residual matrix ***U***, which is a measure of the quality of fit as given by:***I*** = ***WH*** + ***U***

The quality of approximation is determined by the multiplicative update algorithm with 16 replicates of factorizations per data set. Since both the input matrix and the resulting ***W*** and ***H*** matrices are non-negative and the number of solutions can be arbitrarily selected in the algorithm the analysis is greatly simplified and solutions are more unique than those obtained by commonly used singular value decomposition techniques.

## C Results

### Detecting α-helices with site-specific double labels using dihedral indexing

There are many examples of isotope labeled amyloids giving negative frequency shifts.^15^,^30^–^32^,^42^,^44^,^50^–^53^ To test that dihedral indexing can identify α-helical secondary structure as described in the Introduction, we carried out our measurements in conditions that are known to promote and stabilize a helical conformation of hIAPP.

For that purpose, we dissolved hIAPP in 100 mM sodium dodecyl sulfate (SDS) micellar solution, because the structure of hIAPP in SDS micelles has been previously studied using solution NMR spectroscopy and molecular modeling (Protein Data Bank ID: 2L86).^54^ For comparison, we also carried out our measurements in 50% TFE-d5/pH 7.4 TRIS buffer mixture as trifluoroethanol (TFE) is also a very-well known α-helix stabilizing agent.^55^,^56^ The results in TFE are consistent with those measured in SDS micelles and, therefore, we limit our discussion to the latter case only (for TFE results see the ESI†).

**Fig. 2:**
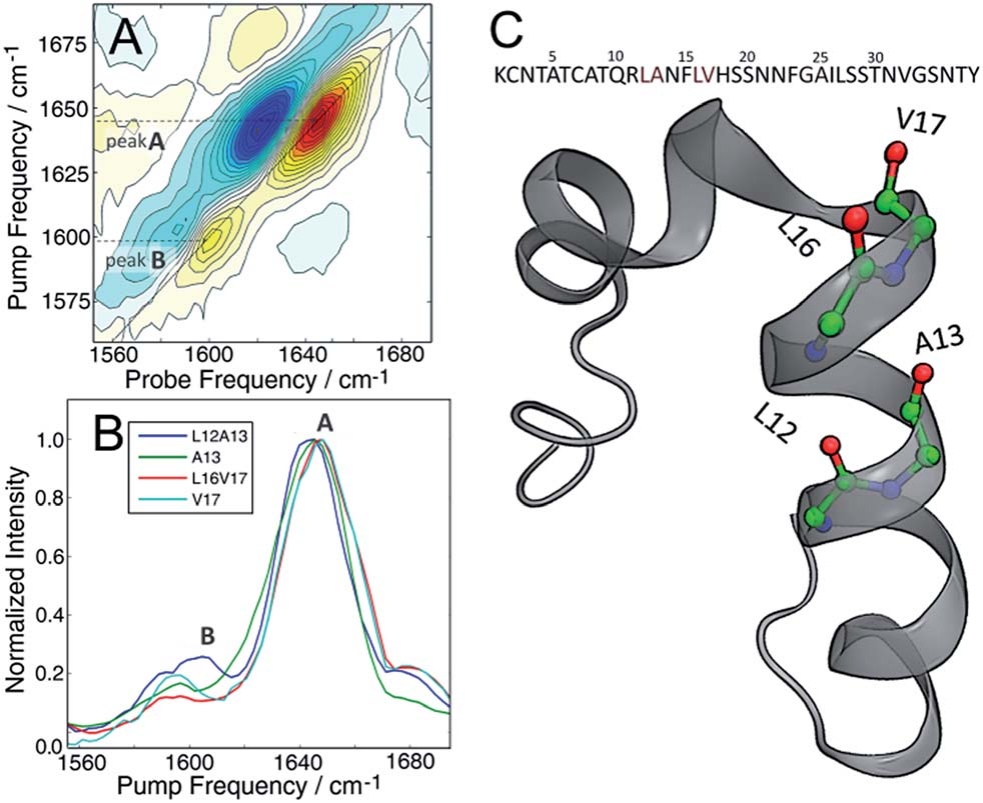
(A) 2D IR spectrum of the L12A13 hIAPP in 100 mM SDS micelle solution. Unlabeled amides give rise to a broad band at 1650 cm^–1^ (peak A). A strong signal from the isotope labels is clearly seen at 1603 cm^–1^ (peak B). (B) Diagonal slices through the *ν* = 1–2 band for all the studied isotope labels measured in SDS micelles. Only the L12A13 label (peak B) shows a strong positive coupling and a great intensity enhancement with respect to the corresponding single label (C) The sequence and NMR structure of human amylin in SDS micelles with the isotope labeling sites highlighted.

The molecular structure of hIAPP in SDS micelles is shown in Fig. 2 with the two sets of double labels highlighted. The L12A13 pair is in the middle of the helix while the L16V17 pair is at the end near a disordered turn. Also shown in Fig. 2 is the measured 2D IR spectrum of the L12A13 hIAPP in SDS micelles (Fig. 2A). The diagonal slices for each peptide are shown in Fig. 2B. The 2D IR spectrum of hIAPP in micelles consists of two amide I bands arising from the unlabeled and the ^13^C^18^O labeled amino acids. The unlabeled residues give rise to a broad pair of peaks at a probe frequency of 1650 cm^–1^, whereas the ^13^C^18^O labeled amide groups create peaks around 1603 cm^–1^. The *ν* = 0–1 transition creates a positive feature on the diagonal while there is a corresponding negative signal originating from the *ν* = 1–2 sequence band transition, shifted from the fundamental band by the anharmonic shift. In previous publications, we illustrated frequencies with diagonal cuts through the fundamental features. In this report, we use cuts through the sequence band. We reach the same scientific conclusions using the overtone, but the data is smoother because there is less spectra congestion with off-diagonal peaks (fundamental cuts shown in the ESI† for comparison). Throughout the paper, frequencies are given with regards to the pump axis unless otherwise specified.

Fig. 2B shows diagonal cuts for L12A13. The maximum of the isotope band is at 1603 cm^–1^, which is 8 cm^–1^ higher than the maximum of the single labeled A13 variant at 1595 cm^–1^. In addition, the L12A13 peak is nearly two-fold more intense, as estimated from the integrated intensities. The simulations (Fig. 1D) predict an 8 cm^–1^ increase in frequency and a two-fold increase in intensity after taking into account that there are two labelled amino acids *versus* one. Thus, the 2D IR data for L12A13 is precisely what is expected for an α-helical structure and fully consistent with the NMR structure. For slices through L16V17, also shown in Fig. 2B, a negligible frequency shift, broadening, and a decrease in intensity is observed for the L16V17 labeled peptide as compared to its V17 counterpart, indicating that the dihedral angles between L16 and V17 are not α-helices and that there is most likely a distribution of structures. Indeed, according to the NMR structure, L16 is at the end of the helix and V17 is at the beginning of a disordered turn (Fig. 2C).^54^ Such a situation will expose L16 and V17 to solvent, creating diagonal disorder in the frequencies, inhomogeneity in the lineshapes, and average the effects of the coupling (the simulations in Fig. 1 are performed with zero diagonal disorder). Indeed, the explicit NMR dihedral angles *ψ* = –30 and *φ* = –80 would have a coupling constant of *β* = +3.1 cm^–1^, although these values are not precise because the turn between the helices is flexible and so the NMR structure is not rigorous in this region. Dihedral angles just 7 deg. different would produce no frequency shift.

Thus, these micelle experiments establish that peptides isotope labeled at two adjacent residues produce positive coupling constants, a frequency shift to higher wavenumbers, and an increase in intensity, if those residues are incorporated into a region of a helix with uniform hydrogen bonding. Disordered regions will have a double labelled peak that is broad due to structural variations. The frequency of labels in disordered regions is less affected by through-bond dihedral coupling. The effects of coupling are more pronounced for ordered structures, for both β-sheets and stacked turns.^33^ An example of negative frequency shifts for β-sheets have been reported previously.^33^ Dihedral indexing is analogous to the NMR method of comparing chemical shifts against a random coil protein to assign secondary structure.^40^ Indeed, the micelle structure of hIAPP was created largely from NMR chemical index measurements.

### Kinetics of hIAPP aggregation using double labels

**Fig. 3:**
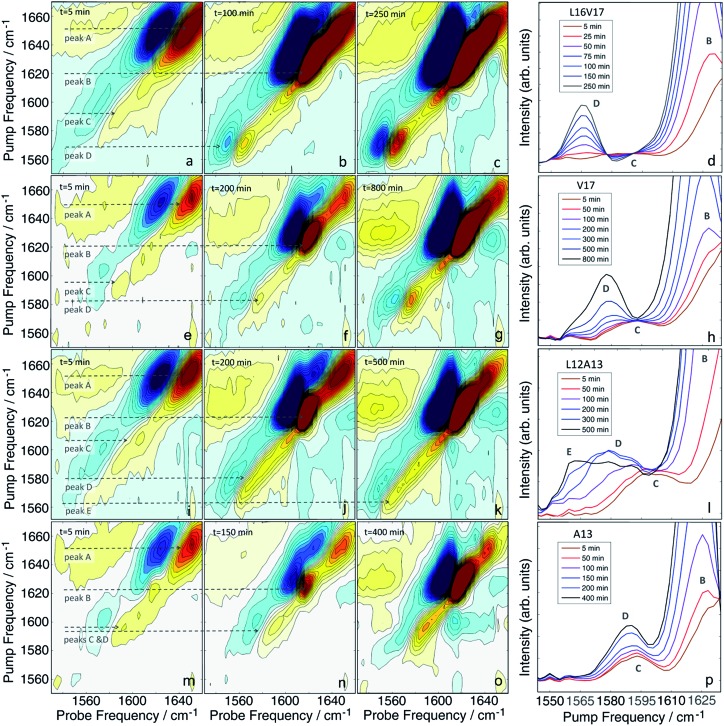
2D IR spectra of L16V17 (a–d), V17 (e–h), L12A13 (i–l) and A13 (m–p) peptides taken at different aggregation times. The time-dependent diagonal intensity slices through the overtone band at different aggregation times are plotted on the right. The most complex intensity changes were observed for the L12A13 hIAPP suggesting that more than two distinct structures are formed during the aggregation process.

Having established that double labels in α-helical regions of a polypeptide exhibit a positive frequency shift in an equilibrated system, we now use double labels to study the kinetics of hIAPP aggregation in aqueous TRIS buffer at physiological pH. As described in Methods, 2D IR spectra are continuously measured during aggregation of hIAPP. The uppermost panels in Fig. 3 show 2D IR spectra of L16V17 at 5, 100, and 250 min during the aggregation time (Fig. 3a–c), chosen to reflect the structures of the monomer, oligomers and fibers. Using oligomer-specific fluorescent microscopy, cross linking, TEM, EPR and 2D IR spectroscopy, it has been previously established that monomers and oligomers exist during these respective periods.^57^–^60^ The diagonal slices through the overtone of each isotope labeled polypeptide are also presented, along with slices from additional times (Fig. 3d). The 1620 cm^–1^ peaks measure the overall β-sheet content of the peptide, while the peaks at 1650 cm^–1^ monitors the native peptide population since these bands are created by the unlabelled peptides. Thus, the kinetics of the 1620 cm^–1^ peak resemble that of a ThT fluorescence experiment.^61^ ThT and TEM experiments are consistent with these observations and are included in the ESI.† At 5 min, the peptide is disaggregated and only exhibits the 1650 cm^–1^ peaks (peak A). At this early time, when the signal is coming from the monomeric species,^58^ the isotope label absorbs at about 1595 cm^–1^ (peak C), is broad 22 cm^–1^ along the diagonal and weak. Hereafter, we call this the “monomer” spectrum. At 100 min, a peak at 1620 cm^–1^ (peak B) appears and the native peak at 1650 cm^–1^ decreases in intensity indicating a progressive aggregation of peptides into amyloid fibrils. At the same time, the corresponding isotope peak of the fiber appears at 1568 cm^–1^ (peak D). At 250 min, the peaks at 1620 cm^–1^ and 1568 cm^–1^ become more intense as the population of amyloid fibers increases. In the kinetic slices, there are no shifts in frequency during aggregation, suggesting that the L16V17 amino acids are following two-state kinetics. By two-state kinetics, we mean that the spectra can be decomposed into that of the monomers plus fibers, with no third conformation needed; a hypothesis that we quantitatively verify below. We do not mean to imply that the kinetics are exponential, which they are not. They are sigmoidal as shown in kinetics plots below.

For comparison, the second panel shows 2D IR spectra of the singly labelled V17 peptide that lacks the dihedral couplings of the double labelled peptide. The 2D IR spectra and kinetics of the unlabelled 1650 cm^–1^ and 1620 cm^–1^ peaks (peaks A and B) are about the same as the doubly labelled peptide, but the isotope labelled region is different. The β-sheet isotope peak is at 1578 cm^–1^ (peak D). It is also narrower and weaker in intensity, consistent with it being one instead of two labelled amino acids. Most importantly, it also appears to follow two-state kinetics, because there are no shifts in frequency, suggesting that each spectrum might be decomposed into a weighted sum of the monomer and fibril spectra.

**Fig. 4:**
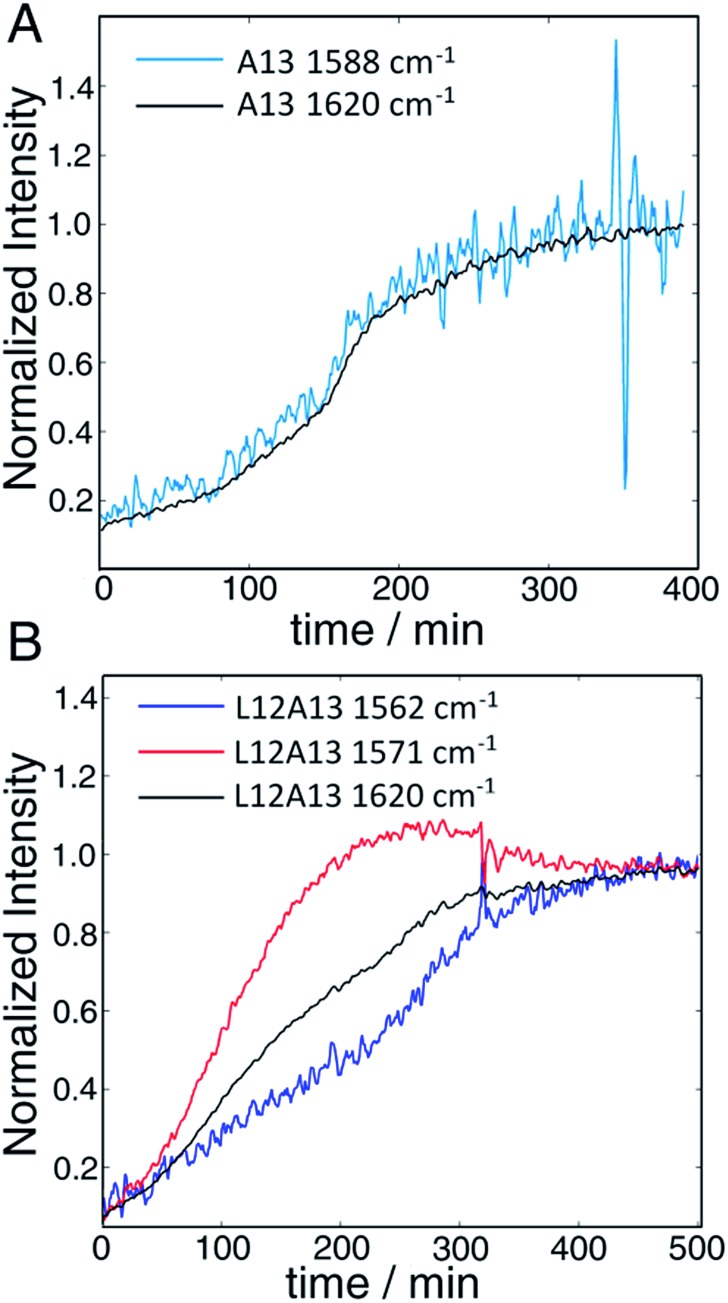
(A) Time-dependent intensity changes measured at the A13 isotope peak maximum overlapped with the kinetics of the main β-sheet peak at 1620 cm^–1^. (B) Kinetics of the L12A13 hIAPP measured at 1560 cm^–1^ and 1571 cm^–1^ compared to the main β-sheet peak at 1620 cm^–1^ (right). The intensity changes of the single label overlap perfectly with that of the main band, whereas the L12A13 shows more complicated kinetics.

The second to last row of figures shows kinetics of the L12A13 peptide. At 5 min, the L12A13 monomer spectrum shows a weak but clearly discernible isotope peak at 1605 cm^–1^ (peak C). At 200 min, a very broad and intense isotope signal appears, which spans a wide range of frequencies with the maximum at pump frequency of 1571 cm^–1^ (peak D). At 500 min, the 1571 cm^–1^ isotope peak decreases in intensity while new intensity appears at 1560 cm^–1^ (peak E). The diagonal slices give a more detailed visualization of the intensity changes (Fig. 4B). The rise and fall at 1571 cm^–1^, along with a new peak at 1560 cm^–1^, indicates that L12A13 is more complicated than simple two-state kinetics. Since isotope labels do not change the protein structure, the differences in kinetics between single and double labels in Fig. 4B indicate that there is a change in coupling.

The final row of spectra show the control experiment using singly labeled A13. In contrast to L12A13, it exhibits two-state kinetics. At 5 min, it has the same monomeric frequency of 1650 cm^–1^, and then a peak at 1588 cm^–1^ growths during aggregation. The frequency difference between A13 and V17 indicates that there are different local electrostatic environment caused by differences in solvation or side chain groups surrounding the amide bond.^33^ Shown in Fig. 4A are the kinetics of the A13 labeled peptide at 1588 cm^–1^ and 1620 cm^–1^, thereby monitoring the isotope label and the fully formed β-sheets of the fiber, respectively. The kinetics are identical, meaning that singly labeled A13 is only monitoring formation of the fiber β-sheets through the couplings between strands, which is what we mean by two-state kinetics. Shown in Fig. 4B are the kinetics for L12A13 at 1562, 1571, and 1620 cm^–1^, monitoring two of the isotope labeled frequencies *versus* the β-sheets of the fiber. The kinetics do not match, and show a clear rise and fall. Since the isotope labels do not alter the structures of the peptides, the spectral differences between A13 and L12A13 labeled peptides can only be caused by differences in coupling. Thus, the fact that they are different, indicates that the dihedral angle between L12 and A13 must be different between the oligomer and the fiber. In other words, the oligomer has a different structure than the fiber β-sheets at L12A13.

### Non-negative matrix factorization

**Fig. 5:**
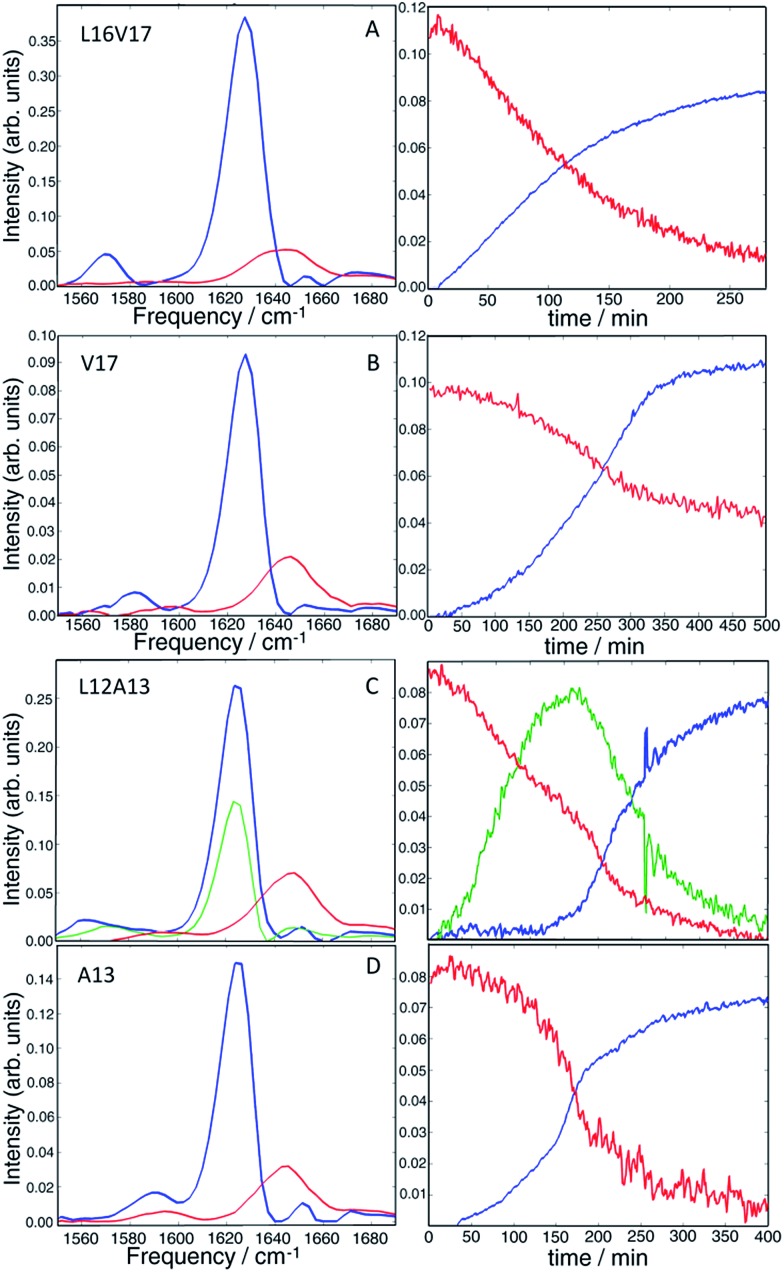
Results of the matrix factorization of the diagonal slices of all the studied isotope labels (A = L16V17, B = V17, C = L12A13, D = A13). The vibrational eigenspectra of monomer (red), fibers (blue) and intermediate (green) are shown on the left, whereas their intensity changes are given on the right.

It is clear from visual inspection of the data that 3 of the 4 samples probably exhibit two-state kinetics and that L12A13 displays at least three-state kinetics. To extract the individual components for each data set, we applied a non-negative matrix factorization (NNMF) algorithm^49^ described in the Materials and Methods section. The results of the NNMF decompositions are presented in Fig. 5.

The left column shows the eigenspectra and the right column the time-dependent intensities for each eigenspectrum. The lag time varies somewhat from experiment to experiment (see ESI† for duplicates), but the conclusions are unchanged. As expected, the data that visually appeared to be two-state kinetics decompose into two separate spectra. Increasing the number of states in the algorithm from 2 to 3 produces a null vector, indicating that only 2 states are unique. The L12A13 spectra fit to a three-state kinetics model. The data cannot be fit using only 2-states and adding a 4^th^ state gives a null vector. Applying the algorithm to just the isotope labelled region below *ω*_pump_ = 1610 cm^–1^, rather than the entire spectrum, produces consistent results which exemplifies that the method is robust (shown in Fig. S5†).

The kinetics of the eigenspectra (the coefficients in the *H*-matrices) are plotted in Fig. 5, which represent populations. For L16V17, V17 and A13, that fit to 2-states, the population of fiber rises sigmoidally, with a corresponding decrease in monomer population. For the three-state kinetics of L12A13, the population of fiber increases sigmoidally, the monomer decays, and the third eigenspectrum grows rapidly during the lag-phase prior to decaying in unison with the population rise of the fibers. Such intensity changes are characteristic of an intermediate species. Thus, we can say with high certainty that the third component corresponds to a transient intermediate identified by dihedral angle changes associated with the L12A13 region that is present during the lag phase of the kinetics.

### Helices

**Fig. 6:**
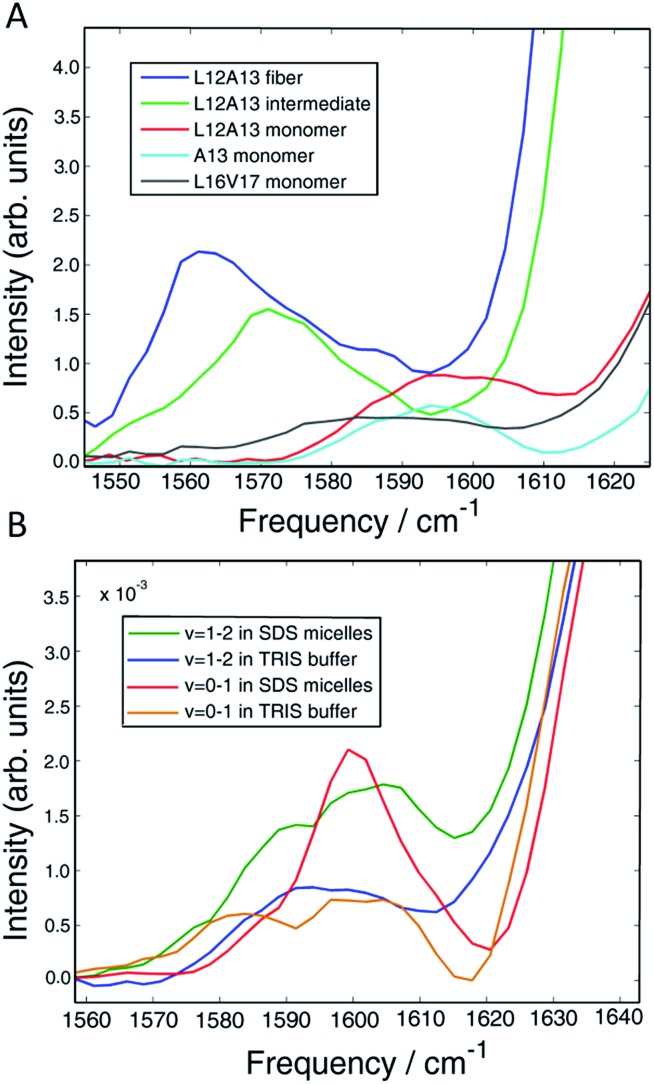
(A) Vibrational eigenspectra of the isotope region of the monomer, intermediate and fiber states of L12A13 as well as the monomeric state of A13 and L16V17 obtained by the matrix factorization method. The intensity above 1600 cm^–1^ observed for the double labeled peptide comes from α-helical conformation of the monomer. (B) Comparison of diagonal slices of L12A13 measured in SDS micelles with the monomer eigenspectra obtained from the data measured in TRIS buffer. The relative intensities show that the population of α-helices in the L12A13 region is in the range of 27–38%.

In Section A, we established dihedral indexing for identifying helical residues. We now analyse the spectra for evidence of α-helix in either the monomer state or oligomer state for hIAPP in buffer with no micelles. Shown in Fig. 6A are reproductions of the data in Fig. 2B for the L12A13 monomer peptide in buffer with an expanded view of the isotope region of the eigenspectra. Also shown is the eigenspectrum for the monomer A13 buffer control experiment originally given in Fig. 5D. The L12A13 monomer eigenspectrum has additional intensity above 1600 cm^–1^ as compared to A13, indicative of a positive vibrational coupling, and therefore, an α-helix at L12A13. Shown in Fig. 6B are slices through both the fundamental *ν* = 0–1 and sequence band *ν* = 1–2 for the monomer in buffer as compared to the SDS micelle control experiments from Fig. 2. We estimate, based on the integrated intensity ratios, that the population of α-helix at L12A13 in buffer is at most 27–38% in the monomeric state. On the other hand, the L16V17 peptide is characterized with a very broad and featureless spectrum in the monomeric state and does not exhibit positive coupling, indicating that it is inherently disordered. Regarding the oligomers, Fig. 6A contains the expanded eigenspectra from Fig. 5C. The eigenspectrum of the intermediate does not show a resolved peak at 1605 cm^–1^ that could be assigned to an α-helix. Rather, there is a minimum at 1605 cm^–1^. If a helix is present, albeit unresolved, not more than 4% of the peptides could contribute. Therefore, we conclude that about 1/3 of the monomer population of hIAPP contains an α-helix at L12A13 in buffer, but that there is no α-helix that we detect at L16V17 in the monomer state nor is there helix in the intermediate oligomeric state at either L12A13 or L16V17 that we detect.

## D Discussion

Our results establish that our method of “dihedral indexing” provides a means for the site-specific detection of α-helices. Through-bond coupling between adjacent amino acids causes their vibrational modes to delocalize, resulting in a frequency shift and intensity enhancement. The shift is positive for α-helices and negative for β-sheets, giving a simple metric for the determination of secondary structure. In principle, single labels report on changes in structure by alterations in environment, but dihedral indexing is much more sensitive to structure than single labels, because it is caused by backbone structure and has the added benefit of intensity enhancement created by vibrational delocalization.

In this section, we discuss three key findings reported here: (1) monomeric hIAPP in buffer is 27–38% α-helical at L12A13, (2) there is no detectable helix at L12A13 nor L16V17 in the oligomeric state and (3) L12A13 exhibits three-state kinetics caused by a dihedral change from the intermediate state to the β-sheets of the fiber. We conclude the Discussion by proposing a structural mechanism that is consistent with these results and other previously reported observations, based on known protein structures of leucine rich repeat proteins.

There are several factors which draw attention to the importance of α-helices in the aggregation of hIAPP. First, the amino acid sequence of the N-terminus of hIAPP suggests that it is amphipathic.^62^ Amphipathicity would promote helix formation on hydrophobic surfaces, such as on bilayers. The N-terminus may be the recognition sequence of hIAPP,^63^ which is a hormone, and, thus, helices may be involved in its function. Second, many molecular dynamics simulations report small helical regions in the N-terminus of the monomer, although the precise nature of the structure depends on force field.^64^ Third, NMR experiments on rat amylin monomers have a short α-helical segment in the N-terminus in a stretch with the same amino acid sequence as human IAPP.^12^,^65^ Fourth, small amounts of HFIP, TFE or DMSO, which are known to stabilize α-helices, accelerate the aggregation.^66^,^67^ Fifth, bilayers also accelerate aggregation, consistent with amphipathicity described above.^13^,^68^ Sixth, NMR experiments on micelles (described above) and electron paramagnetic resonance (EPR) spectroscopy on bilayers, clearly show that hIAPP can adopt helical structure in the presence of lipids.^14^,^54^,^62^ Finally, CD spectroscopy shows that helixes are present throughout the lag time in bilayer experiments, although CD spectroscopy is more ambiguous because CD spectra are sensitive to the length of α-helices and short α-helices show significantly weaker bands, which are hard to detect.^10^

The data shown here establishes that the monomers of hIAPP in buffer are 20–38% α-helical at L12A13. That result is in agreement with MD simulations of unlabeled human amylin and NMR chemical indexing studies on unlabeled rat amylin.^69^,^70^ Replica-exchange molecular dynamics (REMD) simulations on hIAPP predict, besides the random coil, two stable folds of the monomeric polypeptide, one of which is α-helical with a short β-sheet located near the C-terminus. The other structure is a full β-hairpin. The β-hairpin structure was estimated to be more stable than the α-helical conformation by about 0.6 kJ mol^–1^. The calculated abundance of α-helical conformation is 31%, which is in perfect agreement with our experimental estimates.^70^ REMD simulations on rat IAPP predict only α-helical and random coil conformations with relative abundances of 55% and 45%, respectively.^69^ The structure of rat amylin has been studied with solution NMR spectroscopy and the secondary chemical shifts for each residue have been measured.^12^ The NMR secondary chemical shifts predicted by SHIFTX^71^ and SPARTA^72^ algorithms applied to the MD simulations of rIAPP are in reasonable agreement with those experiments. Thus, the methodology used to determine the secondary structures of rat amylin is accurate enough to believe that the structures predicted for the human variant are, indeed, the stable conformations of the monomeric hIAPP observed in our 2D IR experiments.

We find no evidence that the oligomeric intermediate contains helices at either L12A13 or L16V17. It is possible that segments of hIAPP other than L12A13 and L16V17 contain helices, but we think it is unlikely. In micelles, helicity spans C7 to V17 and N21 to S28 residues (Fig. 2C).^54^ In bilayers, it spans A5 to V17 residues and N20 to S23 residues. In MD simulations of the monomer, a helix is found from T9 to V17 residues.^69^,^70^ Helices have not been found in the C-terminus end. Thus, if a helix were present in the intermediate, L12A13 and L16V17 are two of the most likely places for it to reside. The third key finding is the clear observation of three-state kinetics at L12A13. Previous 2D IR studies resolved three-state kinetics in the FGAIL region spanning residues F23 to L27.^30^ Those studies were similar to the kinetics experiments reported here, but used single isotopically labelled peptides. Based on negative frequency shifts, it was concluded that the FGAIL region formed transient β-sheets. Two-state kinetics were observed at A13, and so it was concluded that the well-defined structure of the intermediate did not extend to A13. Based on the results reported here, the previous conclusion regarding A13 is not correct. Two-state kinetics were reproduced in this manuscript (Fig. 5) for singly labelled A13 as previously reported, but three-state kinetics become apparent for when the peptides were doubly labeled with L12A13. Thus, there is a previously unresolved change in dihedral angle during aggregation caused by a difference in structure in the intermediate and fiber states. Based on the negative frequency shifts and intensity enhancement, there must be an increase in transition dipole moment, revealing that the intermediate contains multiple peptides. We do not know the precise structure, but a possible explanation for the negative frequency shift is that L12A13 contributes to a turn or a disordered β-sheet in the intermediate structure, which then rearranges into the parallel β-sheet of the fiber. Indeed, turns in amyloid structures may be more apparent than previously thought, according to recent solid-state NMR structures of Aβ fiber that have many short sections of β-sheet separated by turns to form a tightly packed structure.^74^–^76^

**Fig. 7:**
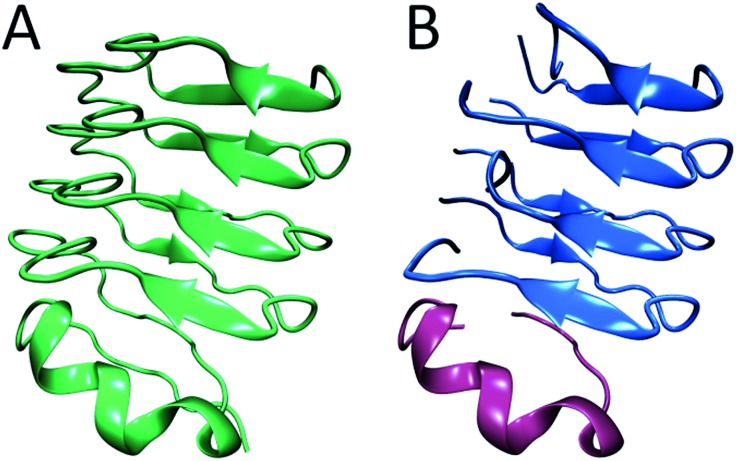
(A) The X-ray structure of BACOVA_04585 leucine-rich repeat protein (pdb: 4FS7) that shows an α-helix on the C-terminus and a long repeating motif of stacked β-sheets and loops formed by a single protein sequence. (B) Structural model of the hIAPP intermediate showing two structured regions separated by a disordered loop, consistent with our 2D IR data. The stacked β-sheets are formed on an α-helical template. The structure is analogous to that of BACOVA protein given on the left, but it is formed by intermolecular assembly of polypeptides instead of intramolecular interactions within a single protein unit. The disulfide bonds in hIAPP are not shown.

In summary, from previous studies we know that hIAPP oligomers contain stacks of parallel polypeptides, with a likely parallel β-sheet in the FGAIL region.^30^,^58^ From our report here, we know that there is a stacked turn or a disordered β-sheet in the L12A13 region and a disordered segment at L16V17 (Fig. 6A). Moreover, we know from our kinetics measurements that the intermediate oligomers are present throughout the lag time, and it is well established that helices catalyze fiber formation and reduce the lag time.^13^,^66^ Thus, is appears that β-sheets are necessary for the stability of the intermediate structure, but that the oligomers can be influenced by helicity. We propose a structure for hIAPP oligomers that is consistent with these observations. The model is structurally heterogeneous, with some polypeptides contributing β-sheet strands and others contributing helices. Our model is based on crystal structures of the leucine-rich repeat proteins. Leucine-rich repeat proteins contain segments of similar sequences of residues that, when the protein is folded, form parallel β-sheets typically 3 or 4 amino acids in length, hence the term “repeat”. They often have helices that stabilize the β-sheets. As an example, residues 298 through 414 of the PDB structure of BACOVA_04585 from Bacteroides Ovatus is shown in Fig. 7A (pdb: ; 4FS7). BACOVA is typical of many leucine-rich repeat proteins. It has a helix at the C-terminus that stabilizes a parallel β-sheet connected by a series of disordered loops and short helices. Many leucine rich repeat proteins are stable, although less so, when their protein is truncated to remove the helix.^73^

Leucine-rich repeat proteins are formed by a single amino acid sequence. We postulate that a similar structure might be formed by an assembly of hIAPP polypeptides. To illustrate this postulate, we present a cartoon of such an intermediate, created by cleaving BACOVA into pieces, as shown in Fig. 7B. The blue peptides contain two short β-sheet segments and a disordered region, such as we have observed at residues 12/13, 23–27, and 16/17, respectively. The purple polypeptide is proposed to be the helix from the monomeric state, which stabilizes the oligomer, but is not itself necessary, analogous to helix stabilization of the leucine-rich repeats.

Unlike most previous models for hIAPP aggregation, which assume that all polypeptides contain the same structure.^30^,^77^–^81^ our oligomer model is structurally heterogeneous, with the majority of hIAPP polypeptides forming stacked β-sheets and turns while other hIAPP polypeptides are involved through (optional) helices.

It has previously been suggested that the amphipathic nature of hIAPP creates oligomers made from helical bundles.^14^,^16^ That mechanism is based on kinetics of hIAPP aggregation in helix-promoting environments like TFE co-solvent and membrane bilayer experiments. However, that model would predict that the population of oligomers, and thus helices, would increase during the lag time, but helix population appears to decrease during the lag time, according to CD measurements.^14^ Moreover, it is not easy to reconcile helical bundles with the three-state kinetics at L12A13 reported here.

We emphasize that we are using the leucine-rich repeat proteins as a conceptual model for hIAPP oligomers, and that much more work is necessary to test our hypothesis. Intermediate structure may depend on concentration, salt, co-solvents, and the presence of vesicles. Moreover, our data does not give information on tertiary structure associations between helices and sheets, and thus we have no direct experimental signature for helix binding. Nonetheless, the proposed structure is consistent with the data presented here and many previously reported results by us and others. First, it is consistent with the known structural features of hIAPP intermediates in the FGAIL region,^30^ reported previously, and the L12A13 and L16V17 regions reported here. Second, it is consistent with the observed three-state kinetics at L12A13 which requires strong couplings between associated peptides – helix bundles cannot have sufficient coupling to explain the experimental features. Third, it accounts for the role of helicity through the stabilization of the oligomers,^10^,^11^,^16^ but does not require high concentrations of helices expected of helix bundling models. In our model, helices act as seeds onto which oligomers form. Thus, small amounts of TFE increase the helicity of monomers and thereby seeds,^67^ would catalyse oligomer formation. Likewise, membranes that promote helix formation of the monomer also create more seeds.^13^ Thus, our model is consistent with the experimental identification of helices in the monomer, but no identifiable helices in the oligomers, because only small concentrations of helices are needed and helices may only be transiently associated with the β-sheet oligomers. Fourth, our model is consistent with kinetic measurements of helix content, which show a steady or declining amount of helix during the lag phase of aggregation (in membrane experiments).^14^ Our model postulates that helicity of monomers seed oligomer formation, and thus the amount of helix is proportional to monomer concentration. Presumably, if helical bundles are driven by amphipathicity, then the helix content would increase during the lag time as more bundles form, which is not observed.^14^ Finally, our model is consistent with experiments by Miranker and coworkers in which polypeptides chemically constrained to be helices were shown to catalyse hIAPP aggregation,^11^ consistent with our seeding mechanism. Thus, a heterogeneous structure model, in which the N-terminus can both adopt an α-helix as well as a stacked β-sheet or turn structure, is consistent with much existing structural data and the new 2D IR data presented here.

## E Conclusions

To obtain the structural data on hIAPP aggregation reported here, we developed a method for monitoring dihedral angles using 2D IR spectroscopy, which we call dihedral indexing. α-Helices *versus* β-sheets give oppositely signed frequency shifts and thus are readily identified by dihedral indexing. By monitoring dihedral angles at L12A13 and L16V17, we established that the monomeric state of hIAPP is 20 to 38% helical at L12A13 (in agreement with NMR and MD simulations), helical features are not observed in the oligomeric state but L12A13 residues exhibit strong coupling between associated polypeptides in the oligomeric state, and that the dihedral angle at L12A13 is not the same in the oligomer and fiber structures, revealing three-state kinetics. We applied dihedral indexing at two locations where it was synthetically straightforward to incorporate ^13^C^18^O double labels which give large (65 cm^–1^) frequency shifts and thereby small background signals. Although background subtraction would be needed, ^18^O labels might be used instead, which can be incorporated anywhere in polypeptides using a simple exchange protocol.^82^ From this structural data, we postulate a mechanism for hIAPP aggregation in which helical monomers seed oligomer formation through structures analogous to leucine-rich repeat proteins. Leucine-rich repeat proteins are stabilized by helices at the end of short β-sheets. We postulate that α-helices seed hIAPP oligomer formation in an analogous manner, with the N-terminus helices of hIAPP monomers helping stabilize small β-sheet oligomers. Although the aggregation mechanism of hIAPP may depend on conditions, such as concentration and the presence of cosolvents or membranes, the proposed mechanism accounts for the residue specific structural information provided here as well as previously published data by us and others. Although far from definitive, it is a working model with specific structural details that can be further tested and refined with 2D IR spectroscopy and other structural biology techniques.

## Conflicts of interest

There are no conflicts to declare.

## Supplementary Material

Supplementary information
