# Supplementary material for: Site-specific detection of protein secondary structure using 2D IR dihedral indexing: a proposed assembly mechanism of oligomeric hIAPP
Source: Chem Sci. 2017 Nov 3;9(2):463–74. doi: 10.1039/c7sc03789a (PMC5868010; doi:10.1039/c7sc03789a)

Electronic Supplementary Material (ESI) for Chemical Science.
This journal is © The Royal Society of Chemistry 2017

Figure S1. (A) 2D-IR spectrum of the pH 7.4 TRIS buffer used in our kinetics measurements. (B) Comparison of the diagonal slices of the TRIS buffer with the slices of hIAPP at two different times since initiation. (C) Subtraction of the Gaussian fit to the background peak at 1540 cm^{-1}.

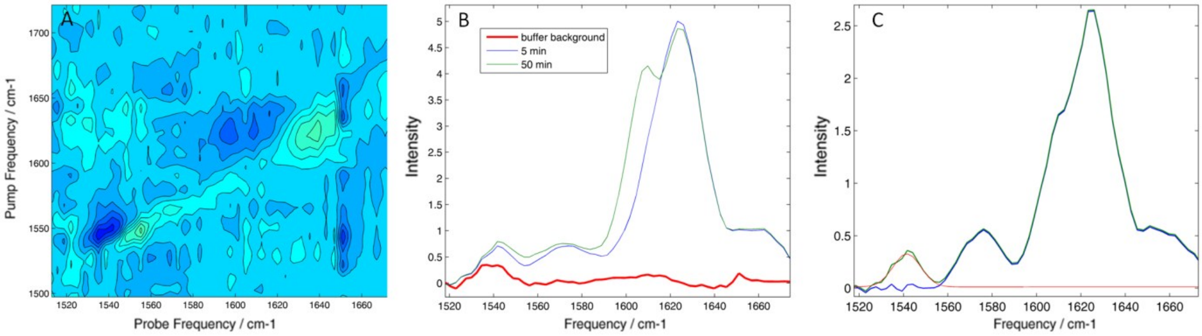

Figure S2. 2D-IR diagonal slices through the overtone of the L12 hIAPP single labeled peptide.

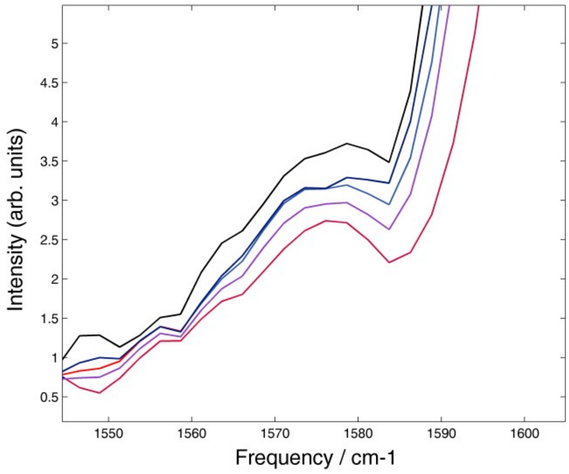

Figure S3. Diagonal slices of all the isotope labeled peptides measured in 50% TFE/50% TRIS buffer solution. Similarly to the case of SDS buffer L12A13 shows the strongest peak indicative of an alpha-helix, whereas other labels show weak and broad peaks.

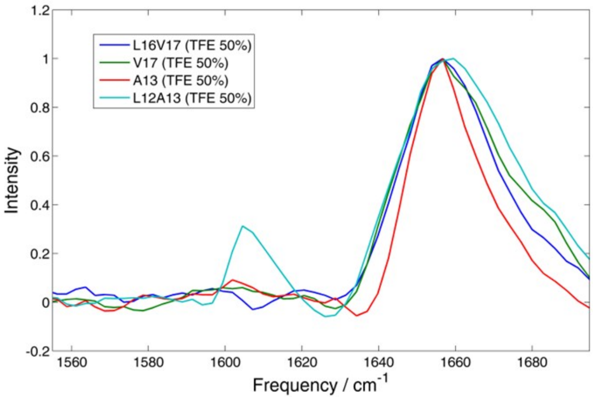

Figure S4. Diagonal cuts through the fundamental band at different aggregation times.

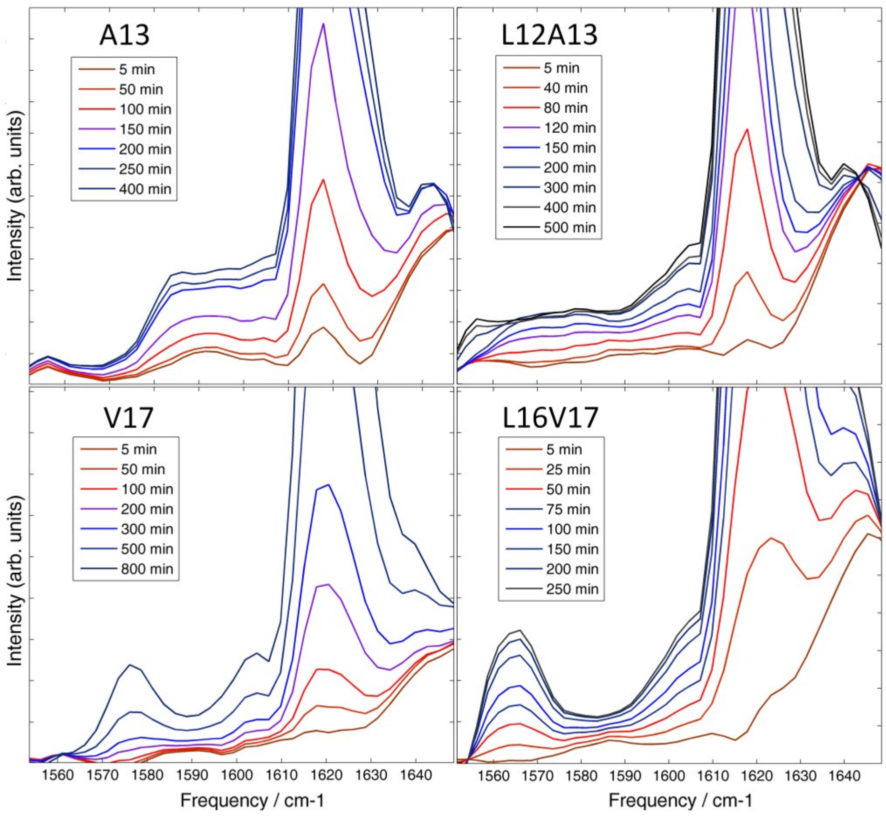

Figure S5. Non-negative matrix factorization of L12A13 in the isotope region only (1540-1610 cm-1)

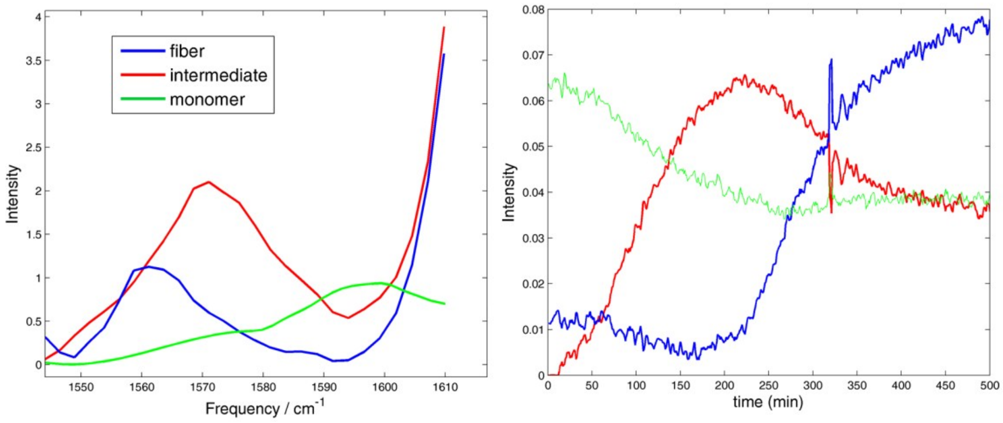

Figure S6. Expanded view of the 2D IR spectra presented in Figure 3 in the main text.

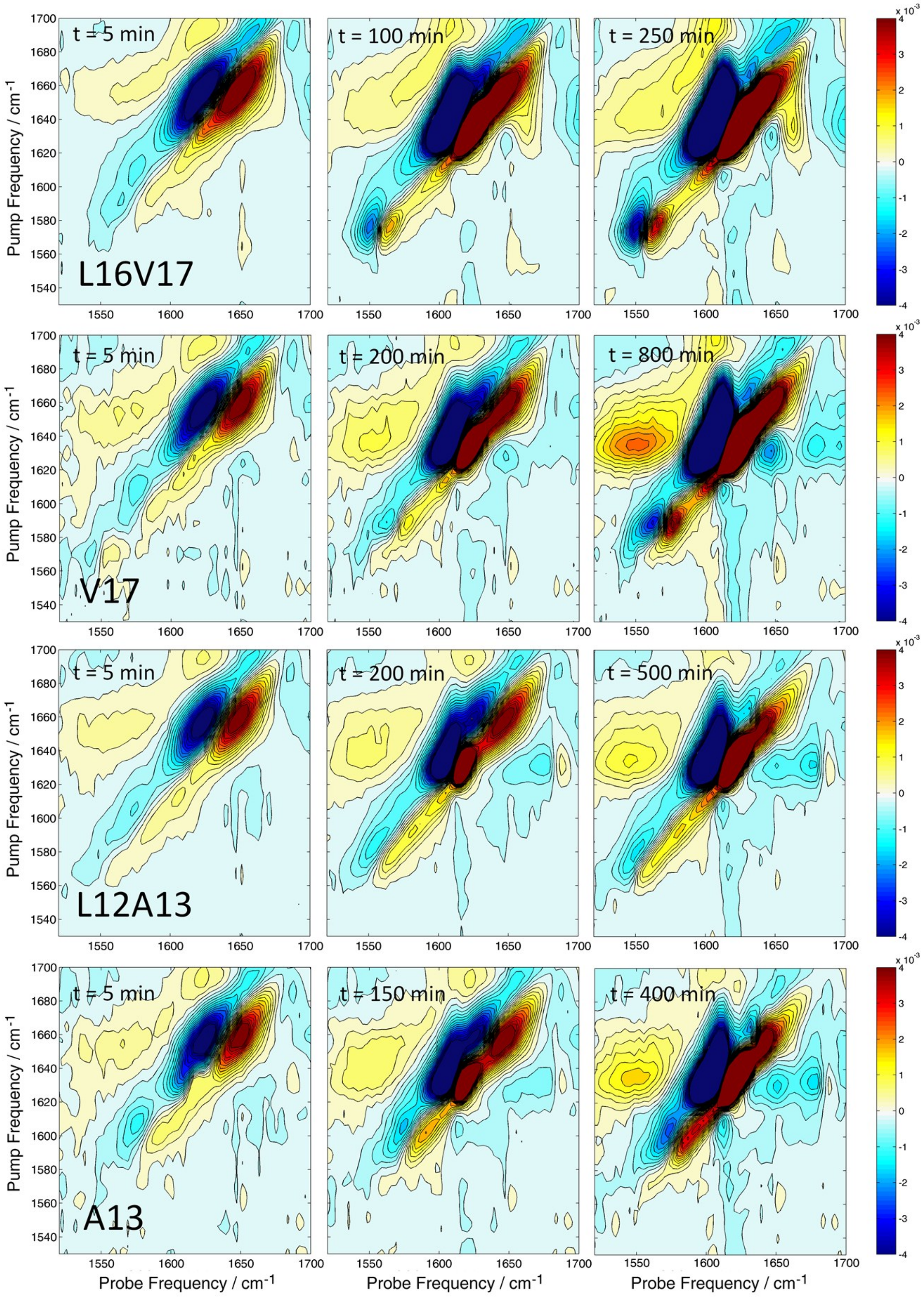

Figure S7. Another data set of L16V17 kinetics exhibiting longer lag phase. The final structure of the fiber is identical to that presented in the main text.

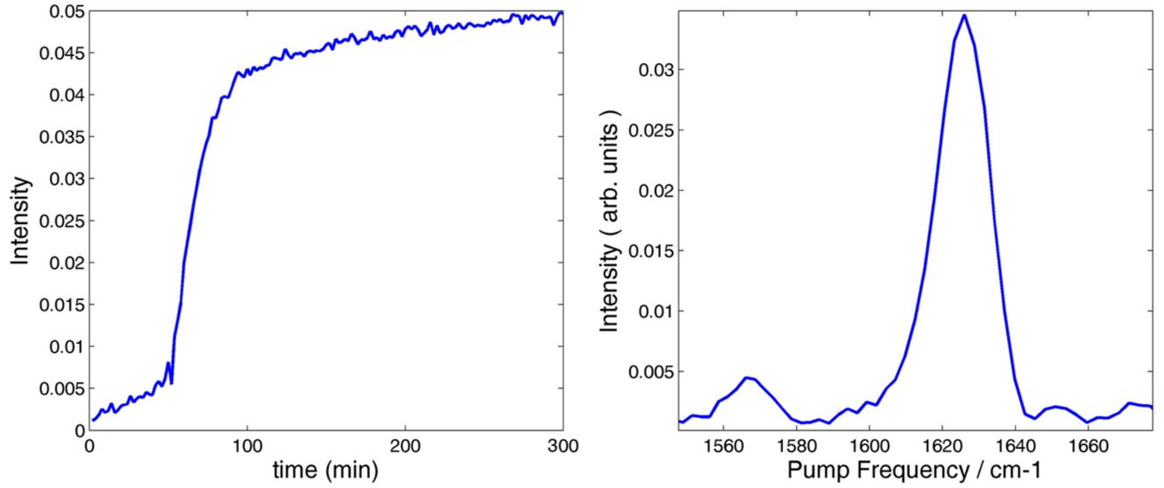

Figure S8. TEM image of 250 μM hIAPP measured at 15 min (lag phase) and 6 hours aggregation times.

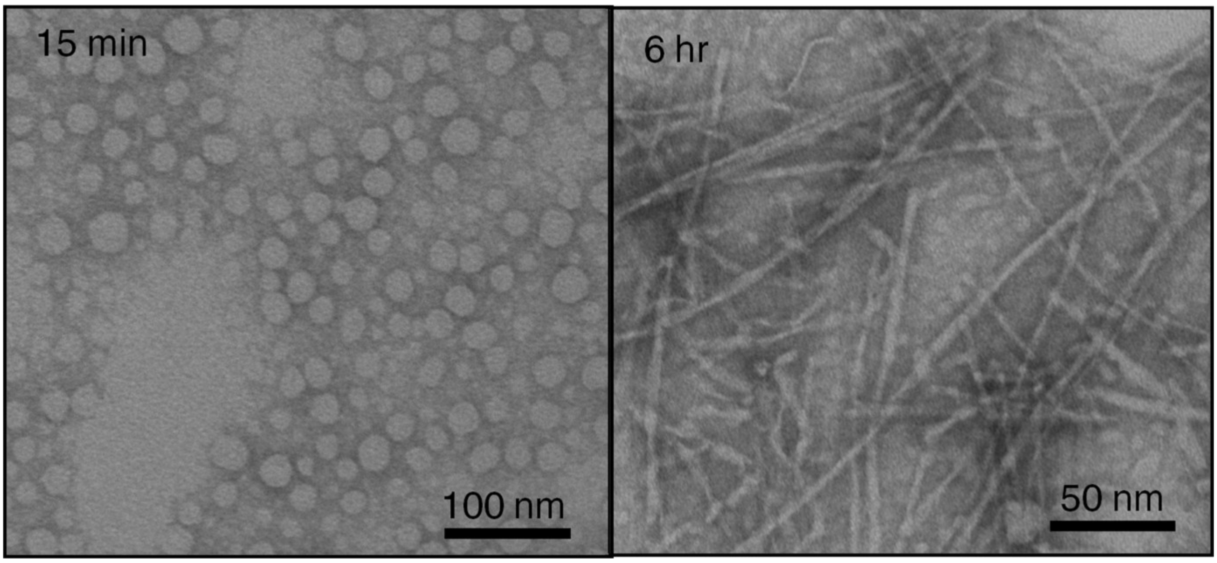

Figure S9. Thioflavin T fluorescence measurement of 0.5 mM L12A13 hIAPP concentration measured in buffer and SDS micelles. The peptide is stable and does not aggregate in SDS solution even after many days of incubation.

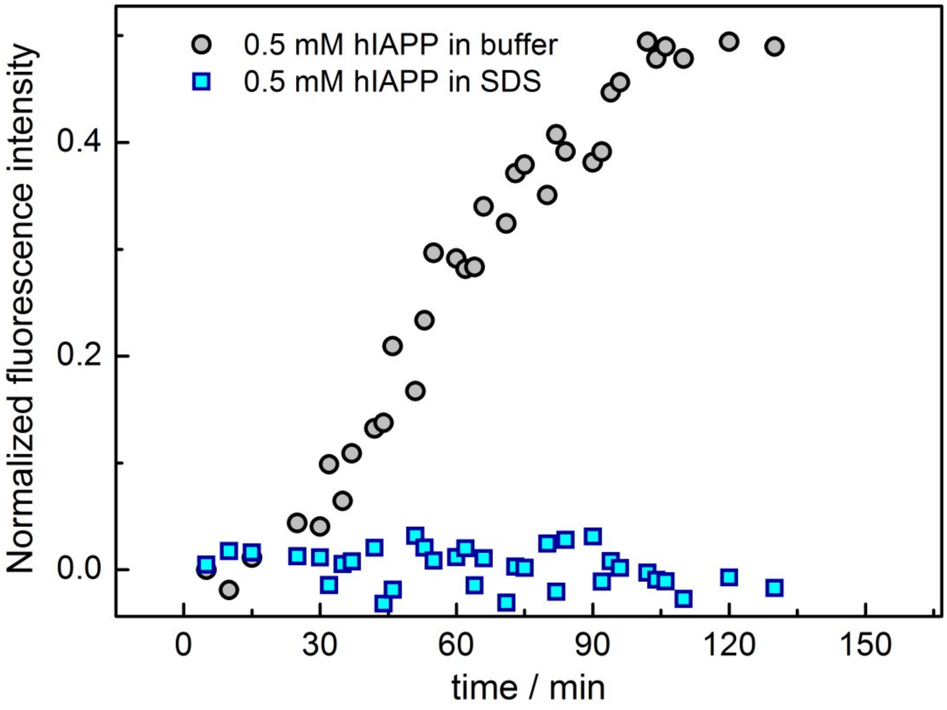

Supplement: Supplementary file 1 [file SC-009-C7SC03789A-s001.pdf]
